# Printable Conductive Hydrogels and Elastomers for Biomedical Application

**DOI:** 10.3390/gels11090707

**Published:** 2025-09-03

**Authors:** Zhangkang Li, Chenyu Shen, Hangyu Chen, Jaemyung Shin, Kartikeya Dixit, Hyun Jae Lee

**Affiliations:** 1 Basic Medical Research Center, Medical School, Nantong University, Nantong 226001, China; 2 Department of Biomedical Engineering, University of Calgary, 2500 University Drive, NW, Calgary, AB T2N 1N4, Canada; 3 Department of Biomedical Engineering, Johns Hopkins University, Baltimore, MD 21218, USA; 4 Department of Mechanical and Manufacturing Engineering, University of Calgary, 2500 University Drive, NW, Calgary, AB T2N 1N4, Canada; kartikeya.dixit@ucalgary.ca; 5 Organization for the Strategic Coordination of Research and Intellectual Properties, Meiji University, 1-1-1 Higashimita, Tama-ku, Kawasaki 214-8571, Japan

**Keywords:** 3D printing, conductivity, hydrogels, elastomers

## Abstract

Printed flexible materials have garnered considerable attention as next-generation materials for bioelectronic applications, particularly hydrogels and elastomers, owing to their intrinsic softness, tissue-like mechanical compliance, and electrical conductivity. In contrast to conventional fabrication approaches, printing technologies enable precise spatial control, design versatility, and seamless integration with complex biological interfaces. This review provides a comprehensive overview of the progress in printable soft conductive materials, with a particular emphasis on the composition, processing, and functional roles of conductive hydrogels and elastomers. This review first introduces traditional fabrication methods for conductive materials and explains the motivation for using printing techniques. We then introduce two major classes of soft conductive materials, hydrogels and elastomers, and describe their applications in both in vitro systems, such as biosensors and soft stimulators, and in vivo settings, including neural interfaces and implantable devices. Finally, we discuss current challenges and propose future directions for advancing printed soft bioelectronics toward clinical translation.

## 1. Introduction

Flexible conductive materials have garnered significant and growing interest in the field of bioelectronics, primarily due to their intrinsic mechanical properties, such as softness, stretchability, and adaptability, which closely resemble those of biological tissues, including resiliency and structural stability under a certain level of pressure [1,2]. Unlike rigid electronic components, these materials can conform intimately to the complex and dynamic surfaces of the human body, including skin, muscles, nerves, and internal organs [3,4]. For example, a magnetic hydrogel octopus was reported with printable hydrogel ink, which has potential in in vivo drug delivery [5]. Another example is about a graphene hydrogel-based electro-bioadhesive interface for in vivo simulation functionalities [6]. A bioadhesive immune-evasive and modulus-matchable brain–machine interface (BMI) was reported based on dopamine-modified poly(3,4-ethylenedioxythiophene) (PEDOT) for in vivo electrocorticography (EcoG) [7]. This mechanical compatibility helps reduce interfacial stress at the interface between devices and biological systems, thereby minimizing immune responses and inflammation while enhancing the long-term stability and biocompatibility of wearable and implantable devices. For instance, the peeling process can evaluate the mechanical compatibility with biological surfaces [8]. As a result, flexible conductive materials are considered highly promising for a wide range of biomedical applications, including physiological signal monitoring, neural stimulation, tissue engineering, and human–machine interfaces [4,9,10].

Traditionally, flexible conductive materials have been fabricated by incorporating conductive fillers into soft polymer matrices [11]. These fillers include metallic nanoparticles, carbon-based nanomaterials, such as carbon nanotubes and graphene, and intrinsically conductive polymers [11]. Through such composite designs, materials can achieve a balance between electrical conductivity and mechanical compliance. In some previous studies about doping methods for the incorporation of conductive fillers into hydrogels, the meticulous control of the ratio of each element can optimize related conditions [12,13]. Although these conventional fabrication strategies have led to important advances, they are typically based on bulk blending and casting methods that lack the spatial precision and design flexibility required for complex biomedical environments. Moreover, maintaining a uniform distribution of conductive fillers and ensuring reliable conductivity under conditions of mechanical deformation continue to present significant challenges. As bulk blending and casting methods, solvent blending is a method including the polymer dissolved in a solvent with the conductive filler to be casted into an intended shape. However, there are disadvantages, such as long hardening time and the difficulty in controlling thickness, resulting in the reagglomeration of the conductive filler [14]. Melt blending is a method according to which the conductive filler is directly put into the molten polymer matrix. In this method, the processing temperature needs to be carefully adjusted to avoid the undesired alteration of mechanical properties, which is related to non-uniformity [15]. Injection molding is a method including molten material injected into a mold to make a desired shape. However, the inaccurate alignment of the conductive filler could negatively affect the electrical properties. Sintering is a method where the mixture of polymer and conductive powders is pressurized and heated below the melting point of the main component until the particles fuse together for an intended network. In this method, uncontrolled porosity after the process could affect mechanical properties. Mechanical deformation can be expressed as structural changes, including stretching, bending, twisting, or cyclic fatigue. It was reported that the mismatch of mechanical properties between conventional biomedical materials and related tissues restricted the utility of the biomedical materials [16]. For the effective analysis of mechanical formation, the relationship between strain and stress is often adopted [17,18].

In response to these limitations, recent years have witnessed the rapid emergence of advanced printing technologies for the fabrication of flexible conductive materials. Techniques such as extrusion-based three-dimensional printing, inkjet printing, and digital light processing have enabled precise control over material placement, layer thickness, and structural geometry [19,20,21]. The overall resolution of extrusion-based three-dimensional printing is around 100 μm [22]. The range of resolution of inkjet printing is between 600 and 1200 dots per inch (DPIs) [23]. In digital light processing, the pixel size, which is directly related to resolution, ranges between 2 and 80 μm [24]. These printing methods allow for the fabrication of complex, high-resolution patterns and provide opportunities for rapid prototyping, design customization, and integration with biological systems, including implants, prosthetics, surgical tools, drug delivery, and tissue engineering. Furthermore, printing approaches are well-suited for direct fabrication on soft tissues or curved surfaces, which is particularly valuable in personalized medicine and regenerative therapies. For instance, in situ bioprinting is beneficial for tissue repair and regeneration, where the effective printing techniques are a key factor to enhance the overall quality of this method [25]. A minimally invasive bioprinting technique was reported for liver regeneration [26].

Among the various categories of soft materials used for bioelectronic applications, hydrogels and elastomers have received particular attention due to their mechanical characteristics and biological relevance [2,27]. To be specific, biocompatibility is a crucial parameter to maintain a stable interface between biomedical devices and related tissues. Biofunctionality is also important because this factor is related to the performance evaluation of biomedical structures. Similarity to native tissue mechanics is an essential parameter to avoid the negative interfacial stress represented by mechanical incompatibility with native tissues. Hydrogels are hydrophilic networks that can retain a large amount of water, offering a tissue-like environment that is favorable for cell interaction and integration [9,28,29]. When functionalized with conductive components, hydrogels can serve as ionic or electronic conductors in bioelectronic devices [30,31,32]. The ionic conduction can be explained by the directional movement of free ions within the hydrogel’s network. As an example, biomineral calcium-ion-mediated conductive hydrogels were reported for ionotropic sensors [33]. On the other hand, the electronic conduction involves the movement of electrons or holes within the conductive network. For instance, the conductive MXene nanocomposite organohydrogel was developed for flexible strain sensors [34]. Elastomers, by contrast, offer excellent elasticity, durability, and resilience, making them suitable for repeated mechanical loading and long-term use in wearable systems [35,36]. Among elastomers, PDMS is the most commonly used elastomer mainly due to its high elasticity and biocompatibility [37]. Polyurethane (PU) is also widely used as a component of implantable devices for its good compatibility with many tissues [38]. Ecoflex is often used in bioelectronics for its low modulus and large stretchability [39]. Polyester elastomers also have biomedical applications owing to biocompatibility, biodegradability, and adjustable mechanical properties [40]. Both types of materials can be adapted into printable formulations, enabling the development of flexible and functional bioelectronic platforms [41].

This review begins by introducing the conventional strategies for fabricating flexible conductive materials, with a focus on conductive filler incorporation into soft polymer matrices. The conductive fillers that can be utilized include carbon-based fillers, such as carbon black, graphene, and carbon nanotubes (CNTs) [42]. Metal nanoparticles, including silver and gold nanoparticles, can also be integrated into soft polymers. Liquid metals, such as gallium-based alloys, or intrinsically conductive polymers represented by polyaniline (PANI) and polypyrrole (PPy), can be used for this purpose as well [43]. It then explores how the development of printing technologies has addressed many of the limitations associated with traditional methods, providing greater spatial resolution, design versatility, and biological integration. The review continues by examining recent advances in printable conductive hydrogels and elastomers, discussing their material formulations, printing techniques, and specific applications in both in vitro systems and in vivo models. Finally, the review identifies the key challenges that remain, including trade-offs between conductivity and mechanical integrity, long-term biocompatibility, material degradation, and regulatory barriers, and offers insights into future directions for advancing the clinical translation and real-world implementation of printed soft bioelectronic devices. As regulatory barriers, rigorous standards, data privacy, and lack of specific guidance can be raised. The inadequate frameworks with regulatory agencies are also reported regarding digital health technologies [44].

## 2. Fabrication of Conductive Soft Materials

**Figure 1:**
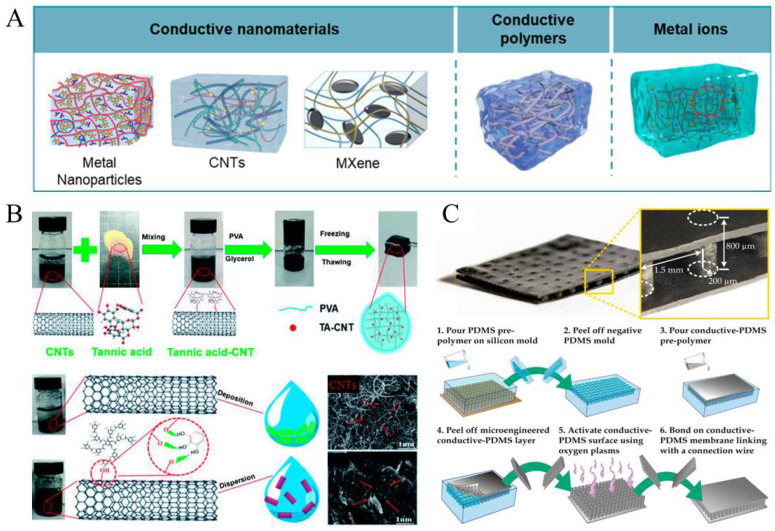
(**A**) Methods for preparing soft conductive materials [11] (copyright © 2024 Cao et al. under the terms of the Creative Commons CC BY 4.0). (**B**) Preparation of conductive hydrogels by incorporating carbon nanotubes [69] (adapted with permission from He et al., copyright 2020 Royal Society of Chemistry). (**C**) Preparation of conductive elastomers using the molding method [27] (copyright © 2015 Hu et al. under the terms of the Creative Commons CC BY 4.0).

Nanoparticles and nanomaterials have garnered substantial interest in recent years due to their unique physicochemical properties and diverse applications in areas such as drug delivery [45,46,47,48,49,50], cancer therapy [51,52,53], cell behavior [54,55,56,57,58], inflammation and bacterial inhibitor [59,60], neuroregeneration [61], and food safety monitoring [62]. In parallel, they also play a pivotal role in the development of conductive soft materials, particularly in the fields of bioelectronics and wearable devices [11,63]. Current fabrication strategies for these materials primarily involve blending conductive components into polymeric matrices (Figure 1A) [11]. This is commonly achieved by incorporating conductive nanomaterials, such as metal nanoparticles, carbon nanotubes (CNTs), and MXenes, into hydrogel or elastomer systems. Metal nanoparticles conduct electricity through free electron movement [11], CNTs enable charge transport via delocalized π-electron systems [32], and MXenes offer high electron mobility along their two-dimensional layers [64,65]. When well-dispersed within soft matrices, these fillers form percolating networks that facilitate efficient electron transport, thereby imparting conductivity to otherwise insulating soft materials. The percolation threshold depends on several factors, such as dispersion, aspect ratio, agglomeration, and surface treatment of conductive fillers [66]. Alternatively, conductive polymers, such as PEDOT:PSS and polyaniline, can be directly mixed with hydrogel or elastomeric substrates to impart electrical conductivity [67]. These polymers possess intrinsic electronic conductivity arising from their conjugated backbone structures, allowing for efficient charge transport. Another effective approach is ionic doping, in which the introduction of ions, such as Li^+^, Na^+^, Fe^3+^, and Al^3+^, into hydrogel networks enhances ionic conductivity. These ions facilitate charge transfer through ion migration under an electric field, making ionically conductive hydrogels particularly attractive for applications in biosensors, actuators, and soft bioelectronics [68]. Notably, certain multivalent ions can also promote crosslinking within the polymer network, thereby improving the mechanical strength and structural integrity of the material. For example, He et al. developed a conductive hydrogel (TA-CNT-glycerol-PVA) by integrating tannic-acid-functionalized CNTs into a polyvinyl alcohol (PVA) matrix dispersed in a water–glycerol mixture (Figure 1B) [69]. Similarly, Hu et al. fabricated biocompatible conductive elastomers by mixing polydimethylsiloxane (PDMS) with conductive nanoparticles, such as acetylene black, followed by molding to produce microengineered conductive elastomeric electrodes (Figure 1C) [27]. These conventional fabrication techniques are well-documented in the literature and have demonstrated reliable performance across various applications.

However, these methods often lack the capability for precise spatial control and complex structural customization, which limits their utility in emerging biomedical applications. Several limitations exist in terms of scalability, uniform dispersion, and difficulty in mass production. In this context, 3D-printing technologies offer a powerful alternative, enabling programmable fabrication with high resolution, tunable architecture, and seamless integration of multifunctional materials [70].

## 3. Significance of Printing Techniques

Although significant progress has been made in the development of conductive soft materials, their widespread implementation in real-life applications still faces considerable challenges. At present, most conductive soft materials used in daily life rely on conventional micro- and nano-fabrication techniques, which often limit their accessibility and scalability. The emergence of 3D printing and other advanced printing techniques has provided a more practical and versatile approach, enabling these materials to be more easily integrated into everyday technologies, particularly in biomedical applications [71]. In this context, printing facilitates the fabrication of biocompatible, patient-specific devices, such as wearable sensors, implantable electrodes, and tissue scaffolds, which require both mechanical compliance and precise structural control [72].

**Figure 2:**
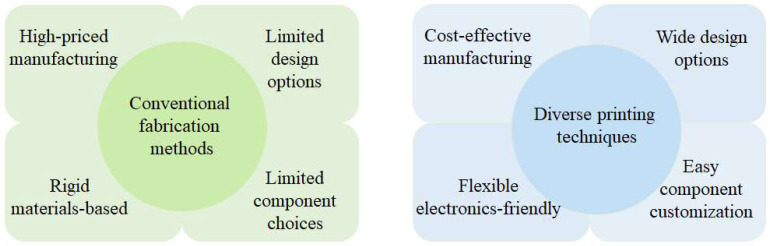
Comparison between conventional fabrication methods and diverse printing techniques.

The introduction of printing techniques into conductive flexible materials has several advantages, which mainly contributes to the development of a wide range of wearable and flexible electronic devices for numerous applications (Figure 2). Firstly, printing techniques are often cost-effective with respect to other conventional fabrication methods, such as microfabrication techniques, usually including deposition, patterning, and etching [41,73,74]. These techniques often require multiple huge equipment, related chemicals, and their treatments, which causes the increase of the total costs to fabricate intended structures [75,76,77]. Compared to the microfabrication techniques, the printing techniques have a variety of printing techniques combined with several types of filaments or inks for conductive flexible materials, which leads to the revolutionary reduction of related costs.

Secondly, printing techniques are more conducive to flexible devices than the conventional fabrication methods due to the use of compliant substrates. Recently, a number of filaments or inks with conductive and flexible properties are easily available [63,78,79,80]. The selection of suitable printing materials with adapted printing techniques enables the researchers to fabricate flexible devices as they want.

Thirdly, printing techniques extend customized design options using conductive flexible materials, such as carbon-based materials [81]. Although the aforementioned microfabrication techniques are known for precise and fine processing with the aim of micro- or nano-structures, printing techniques are being improved with advanced technologies in order to compensate their previous disadvantages.

Fourthly, printing techniques increase the number of options for component materials. For example, the equipment for the microfabrication methods is optimized for commonly used materials, which makes it difficult to attempt novel combinations of materials. However, with current printing techniques, when we focus on conductive flexible materials, it is possible to select commercial products or to produce customized filaments using a mixture of proper pellets to enhance electrical conductivity and structural elasticity [82,83,84,85]. The various combinations of pellets enable the researchers to optimize their own filaments for personalized purposes.

For these reasons, various printing techniques are being developed for biomedical applications [86,87]. For example, fused deposition modeling (FDM) consists of thermoplastic filaments that are heated and deposited in an intended shape. This technique can adopt a wide range of materials with high printing speed. Another printing technique is stereolithography (SLA), which is realized with photopolymerization. This method is known for high accuracy, although the parameters of photopolymers need to be carefully adjusted. Selective laser sintering (SLS) is a printing technique based on the laser as a power source, showing high resolution. Inkjet 3D printing is layered manufacturing technology, where polymeric bioinks can be used for biomedical applications.

## 4. Application of Printed Conductive Elastomers In Vitro

Among conductive soft materials, conductive elastomers stand out due to their unique combination of electrical conductivity and mechanical flexibility, making them ideal candidates for biomedical applications where conformability to soft tissue and long-term biocompatibility are required. When combined with emerging fabrication technologies, such as 3D printing, these materials can be precisely patterned into complex geometries that support functional integration with biological environments. Combining conductive elastomers with precise fabrication strategies allows for applications in both in vitro and in vivo settings. We first examine in vitro applications, particularly the development of wearable devices, flexible bioelectronic systems, and soft interfaces for physiological monitoring.

**Figure 3:**
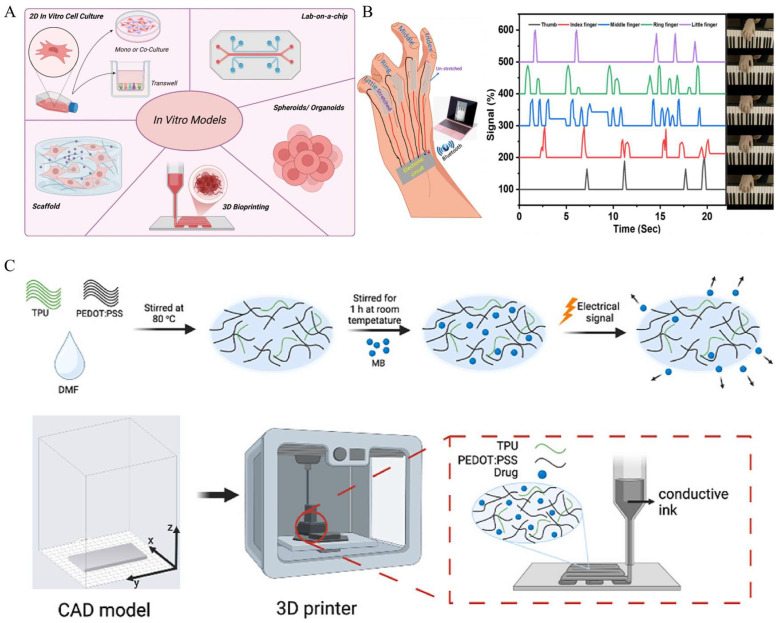
(**A**) Application of in vitro models using conductive biological materials [93] (copyright © 2025 Serafrin et al. under the terms of the Creative Commons CC BY 4.0). (**B**) Ionic conductive elastomer-based full-hand sensor integrated with Bluetooth communication [94] (adapted with permission from Borayek et al., copyright 2022 American Chemical Society). (**C**) 3D printing of conductive ink for in vitro drug release [35] (copyright © 2024 Alkahtani et al. under the terms of the Creative Commons CC BY 4.0).

In vitro studies are defined as scientific research on biological components outside a living organism and under artificially controlled environments. The role of materials consisting of these controlled conditions is crucial to clarify the behaviors of biological components represented by cells, tissues, and other biological molecules. To create highly controlled environments with affordable materials, printing technologies can be an effective option compared to the conventional fabrication methods. For this reason, printed conductive elastomers are often utilized in in vitro studies, such as for tissue engineering (while the final application is intended for in vivo implantation, most of the current experimental work remains at the in vitro stage) [88]. As conductive polymers, polypyrrole (PPy), polyaniline (PANI), and poly(3,4-ethylenedioxythiophene) (PEDOT) were reported owing to their high electrical conductivity and biocompatibility [89]. These conductive elastomers can be applied to scaffolds for cell growth and tissue regeneration [90,91]. Hashemi et al. developed a 3D-printed scaffold for its in vitro evaluation in enhanced angiogenesis using a conductive biomaterial ink composed of chitosan and polyaniline [92]. The electrical characteristics of 3D-printable materials can be a useful factor to understand the electrical environment of tissues and its effect on cell migration, proliferation, and differentiation. Figure 3A shows examples of the application of printed conductive elastomers in in vitro studies. The combination of in vitro studies and printing techniques has the potential to establish sophisticated in vitro models, such as organ-on-a-chips, as described in Figure 3A [93]. The application of biodegradable conductive materials, such as carbon-based nanocomposites, with bioprinting techniques is believed to promote sustainable biomedical research, minimizing negative environmental impacts. Borayek et al. suggested a wearable device using ionic conductive elastomers and 3D-printing techniques (Figure 3B) [94]. This study stresses the achievement of the near-zero hysteresis behavior of the ionic conductor, which can improve the accuracy and reliability for in vitro sensing applications. Another example presents electrode fabrications for drug delivery [35]. The combination of 3D-printing techniques and conductive polymers based on PEDOT:polystyrene sulfonate (PEDOT:PSS) demonstrated an electro-responsive system for effective in vitro drug release (Figure 3C). Given that in vivo studies, to be mentioned below, often raise ethical issues due to the treatment of living organisms, in vitro studies are often adopted to evaluate cytotoxicity in terms of drug development. Printed conductive elastomers can also be transformed into certain kinds of electrochemical sensors [95].

## 5. Application of Printed Conductive Elastomers In Vivo

**Figure 4:**
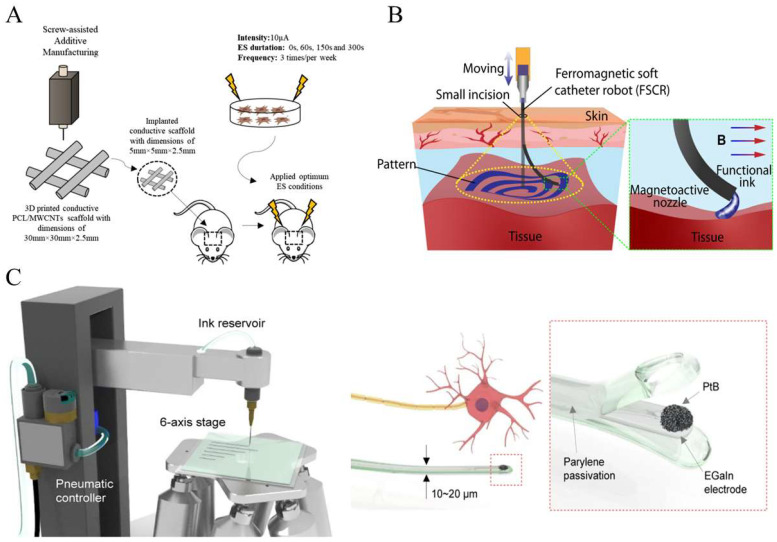
(**A**) In vivo exogenous electrical simulation process [96] (copyright © 2021 Silva et al. under the terms of the Creative Commons CC BY 4.0). (**B**) In vivo minimally invasive bioprinting [97] (copyright © 2021 Zhou et al. under the terms of the Creative Commons CC BY 4.0). (**C**) Printing system of a liquid metal and the comparison between the soft neural probe and neuron [98] (copyright © 2024 Park et al. under the terms of the Creative Commons CC BY 4.0).

In vivo studies aim at scientific discoveries following a series of experiments conducted inside a living organism. In this case, the biological components are not isolated but capable of interacting with other parts of the living organism, which is a key difference from in vitro studies. Even though meticulous ethical considerations are required compared to in vitro studies, in vivo studies are better suitable to comprehend the overall perspective of the nature, consisting of targeted biological components and their surroundings. For these objectives, printed conductive elastomers were developed as multiple types of medical devices, including implantable sensors, artificial muscles, and actuators [36,43,72]. Compared to the aforementioned in vitro studies, these materials need to be more biocompatible to easily integrate with the biological environment of a living organism and be adapted for monitoring necessary biological signals. In addition, contrary to in vitro studies, the conductive elastomers used for in vivo studies need to ensure long-term durability in the living organism, where many chemicals are associated with each other. At the same time, these materials are supposed to bring minimized side effects on the complex biological system of the living organism. Figure 3 introduces the application of printed conductive elastomers for in vivo studies. E Silva et al. elaborated biodegradable polycaprolactone (PCL) and multi-walled carbon nanotubes (MWCNTs) for the in vivo study of 3D-printable conductive materials for electrical stimulation (Figure 4A) [96]. The use of PCL/MWCNTs for electrical stimulation affected a meaningful bone tissue formation and angiogenesis. As an in vivo bioprinting technique, Zhou et al. reported ferromagnetic soft catheter robots (FSCRs) for bioprinting inside the human body using conductive polymers with a combination of silver flakes and alginate solution (Figure 4B) [97]. The FSCR system was found to realize stable ink extrusion with various printing materials on the planar surfaces of natural organs. Park et al. showed the in vivo integration of eutectic gallium-indium (EGaIn)-based soft neural probes for its conformal inclusion in the body and its effective delivery of neural signals to a smartphone (Figure 4C) [98]. This device includes the direct printing of subsidiary electronics on the cranial surface. Dong et al. suggested screen-printed electrode arrays for in vivo neural recording using polydimethylsiloxane (PDMS) substrates and EGaIn [99].

## 6. Application of Printed Conductive Hydrogels In Vitro

**Figure 5:**
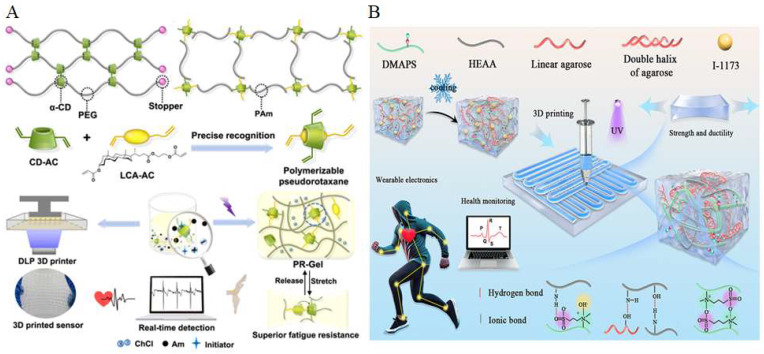
(**A**) Preparation of conductive hydrogel wearable devices by the DLP (digital light processing) method [31] (copyright © 2023 Xiong et al. under the terms of the Creative Commons CC BY 4.0). (**B**) Preparation of conductive hydrogel wearable devices by the extrusion method [110] (adapted with permission from Liu et al., copyright 2020 Elsevier).

Hydrogels are three-dimensional polymeric networks capable of retaining large amounts of water, offering excellent softness, biocompatibility, and tunable mechanical properties [100,101,102,103]. Compared to elastomers, hydrogels more closely mimic the mechanical properties of biological tissues, making them particularly advantageous for applications involving direct contact with the skin or biological interfaces [104,105,106,107]. Moreover, their intrinsic ionic conductivity and permeability to biomolecules enable seamless integration with biosensing and bioelectronic systems. For in vitro applications, hydrogel-based conductive materials have been widely explored in the development of wearable devices. These hydrogel wearables benefit from their superior conformability, breathability, and skin-friendliness, which allow for long-term, comfortable wear and reliable signal acquisition [4,30]. Additionally, the ability to incorporate sensing, adhesion, and self-healing functionalities into hydrogel systems further enhances their potential in next-generation wearable electronics [108,109]. A representative example is the recently developed polymerizable rotaxane hydrogel (PR-Gel), which integrates topological design with photopolymerizable chemistry, as shown in Figure 5A [31]. The PR-Gel was synthesized by assembling a polymerizable pseudorotaxane crosslinker through precise host–guest recognition between acrylated β-cyclodextrin (CD-AC) and an acrylated lithocholic acid derivative (LCA-AC). This supramolecular crosslinker was mixed with acrylamide (Am), choline chloride (ChCl), and a photoinitiator, followed by UV-initiated photopolymerization to form a topological hydrogel network. The resulting PR-Gel exhibits excellent stretchability, fatigue resistance, and printability via digital light processing (DLP) 3D printing, enabling its application in real-time wearable sensing. The incorporation of polymerizable carbon–carbon double bonds ensures photocurability and enables precise 3D printability, while the introduction of topological crosslinks imparts the hydrogel with excellent mechanical resilience. In addition, the presence of choline chloride (ChCl) confers ionic conductivity, making the material suitable for real-time biosignal detection in wearable electronic applications. This approach highlights the potential of topologically engineered hydrogels in next-generation wearable electronics, particularly through their compatibility with high-resolution 3D printing. Extrusion-based printing is another widely adopted technique for fabricating conductive hydrogel-based wearable devices. Liu et al. proposed a novel double-network (DN) hydrogel strategy to develop a tough, adhesive, conductive, and injectable material suitable for extrusion processes (Figure 5B) [110]. In this system, the first network is constructed from agarose macromolecules, forming a rigid polymer matrix that effectively resists deformation under gravity and exhibits high-yield stress following a thermally induced sol–gel transition. The second network consists of a flexible copolymer composed of [2-(methacryloyloxy)ethyl]dimethyl-(3-sulfopropyl) ammonium hydroxide (DMAPS) and N-hydroxyethyl acrylamide (HEAA). This zwitterionic polymer network offers strong hydration capacity and engages in multiple noncovalent interactions, such as ionic and hydrogen bonding, thereby improving the shear-thinning properties of the precursor solution and facilitating smooth extrusion through the nozzle. Following the printing process, the hydrogel is cured via ultraviolet (UV) light-induced radical polymerization, using Irgacure 1173 (I-1173) as the photoinitiator. The extensive hydrogen bonding between the two interpenetrating networks further enhances the mechanical strength and ductility of the printed hydrogel. Moreover, the presence of zwitterionic groups in the polymer matrix establishes ion migration pathways, which significantly contribute to ionic conductivity. These features make the resulting hydrogel particularly attractive for applications in bioelectronics and wearable sensing technologies.

## 7. Application of Printed Conductive Hydrogels In Vivo

**Figure 6:**
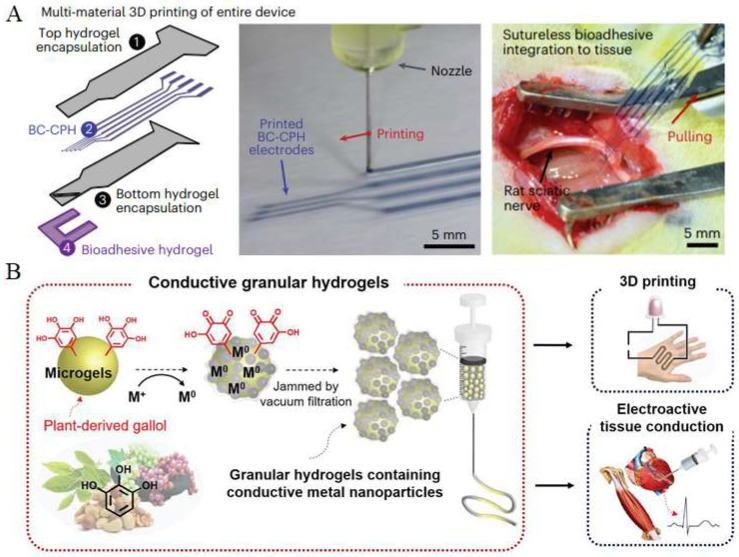
(**A**) Schematic illustration (left) and image (middle) of multi-material 3D-printing-based fabrication of an all-hydrogel bioelectronic interface, and image (right) showing sutureless, robust bioadhesive integration of the all-hydrogel bioelectronic interface on a rat sciatic nerve [10] (adapted with permission from Zhou et al., copyright 2022 Springer Nature). (**B**) Overall schematic of printable conductive granular hydrogels inspired by plant-derived gallol and used for biomedical applications [111] (copyright © 2019 Shin et al. under the terms of the Creative Commons CC BY 4.0).

The excellent biocompatibility of hydrogels renders conductive hydrogel materials suitable for implantation in vivo. The incorporation of 3D-printing technologies enables the precise fabrication of conductive hydrogel architectures, allowing for better adaptation to the complexity of physiological environments, which can be represented by the situation where thin and tiny morphologies of tissues need to be carefully taken into account to prevent any negative effects on tissues, followed by the installation of biomedical materials or devices. For example, Zhao et al. developed a conductive bioink, termed BC-CPH, consisting of a phase-separated mixture of poly(3,4-ethylenedioxythiophene):polystyrene sulfonate (PEDOT:PSS) as the conductive component and hydrophilic polyurethane as the structural matrix (Figure 6A) [10]. This bioink exhibits both high electrical conductivity and excellent mechanical resilience. Through extrusion-based printing, patterned BC-CPH circuits were directly fabricated and encapsulated within a hydrogel matrix to construct fully hydrogel-based bioelectronic interfaces. The incorporation of a bioadhesive layer enabled rapid, robust, and suture-free integration with biological tissues, promoting conformal contact while minimizing mechanical mismatch and immune response. This strategy presents a promising route toward the development of implantable bioelectronic systems. Similarly, Shin et al. introduced an innovative approach for constructing injectable and conductive hydrogels by assembling hyaluronic-acid (HA)-based microgels into granular architectures featuring metal–phenolic coordination networks (Figure 6B) [111]. Electrical conductivity was achieved via in situ metal reduction, mediated by gallol moieties, which are naturally occurring polyphenols capable of reducing metal ions to form conductive nanoparticles. Compared to conventional nanoparticle embedding, this in situ strategy provides improved electrical performance and mechanical integrity. Additionally, the granular hydrogels exhibit excellent injectability and shear-thinning behavior, making them well-suited for 3D printing of complex electroactive constructs. These materials show strong potential for in vivo applications, particularly as bioelectronic interfaces for electrically active tissues, such as myocardium and skeletal muscle. These studies highlight the critical role of advanced 3D-printing techniques in enabling the precise fabrication of multifunctional, tissue-integrated bioelectronic systems.

## 8. Discussion

**Figure 7:**
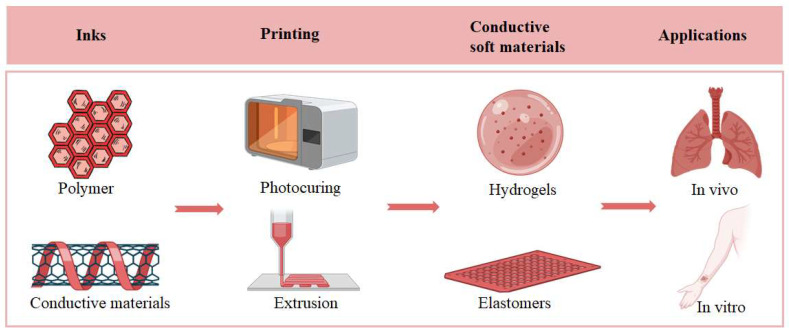
Schematic diagram of preparing printable conductive hydrogels and elastomers.

Printable conductive hydrogels and elastomers have emerged as key materials for biomedical applications, including tissue engineering, biosensors, and soft bioelectronics. The fabrication of these materials typically involves incorporating conductive components into hydrogel or elastomer precursors, followed by shaping them through advanced printing techniques. This process requires precise control over the formulation and rheological properties to ensure smooth printing, rapid solidification, and structural fidelity. Common strategies include blending conductive fillers, such as carbon nanotubes, graphene, or metallic nanoparticles, into polymer matrices, or introducing conductive polymers like polyaniline (PANI), polypyrrole (PPy), or poly (3,4-ethylenedioxythiophene) (PEDOT) to establish efficient electrical pathways while maintaining mechanical compliance and biocompatibility. At present, the mainstream approach to fabricating these materials involves loading conductive components into the hydrogel or elastomer precursor and then processing them through either extrusion-based printing or photopolymerization-based printing (Figure 7). In addition to these conventional approaches, printing technologies have continued to advance, and two emerging strategies have shown distinct advantages. The first is in situ printing, which allows direct deposition of the conductive material at the target site (Figure 4B), minimizing the need for post-print manipulation and enabling rapid clinical adaptation. The second is encapsulation printing, in which conductive components are encapsulated within the hydrogel or elastomer matrix during printing (Figure 6A), allowing faster and more reliable integration of functional materials for biomedical use. These methods significantly enhance the convenience and efficiency of introducing conductive materials into the human body.

The design of printable conductive hydrogels and elastomers must satisfy several essential requirements, including printability, biocompatibility, mechanical flexibility, and electrical conductivity suitable for the intended application. Printability requires that the material possesses suitable rheological properties, such as shear-thinning behavior for extrusion-based printing, or sufficient crosslinkable groups (e.g., carbon–carbon double bonds) for photopolymerization, to ensure smooth deposition, shape fidelity, and structural stability after printing. Flexibility is particularly important for matching the mechanical properties of soft tissues, while incorporated conductive fillers provide the electrical functionality. Moreover, ensuring biocompatibility is critical, as these materials are often intended for direct contact with living tissues and must maintain cell viability, minimize immune response, and exhibit long-term stability in physiological environments. In the current research landscape, there is no strict pursuit of maximizing flexibility or conductivity after printing. Instead, these properties are optimized based on specific application scenarios. For example, a hydrogel-based electrode applied to the surface of a beating heart only needs to generate stable electrical signals for real-time monitoring rather than achieving the highest possible conductivity or stretchability. Hydrogel matrices commonly used for these applications include poly(vinyl alcohol) (PVA), polyethylene glycol (PEG), alginate, gelatin, hyaluronic acid, and gelatin methacrylate (GelMA), all of which provide a water-rich environment and tunable physicochemical properties. Elastomeric matrices, such as polydimethylsiloxane (PDMS), polyurethane (PU), and silicone-based networks, are frequently employed due to their inherent softness and stretchability. By carefully balancing these factors, such as printability, biocompatibility, mechanical compliance, and electrical performance, researchers are developing advanced conductive hydrogels and elastomers that hold promise for next-generation biomedical technologies, including implantable bioelectronic devices, functional tissue scaffolds, and real-time physiological monitoring systems.

Future research on printed conductive hydrogels and elastomers should focus on improving printing precision to enable complex and patient-specific designs. Enhancing long-term stability under physiological conditions and achieving a better balance between printability, flexibility, conductivity, and biocompatibility are also critical. Additionally, integrating functionalities, such as self-healing and biodegradability, along with establishing standardized evaluation methods, will be key to advancing clinical applications.

## 9. Conclusions

Printable soft conductive materials represent a significant advancement in the field of bioelectronics, as they uniquely combine mechanical compliance, electrical functionality, and fabrication adaptability. Among soft conductive materials, hydrogels and elastomers constitute two major classes with complementary characteristics. Hydrogels provide a hydrated and biocompatible environment suitable for cell interaction and integration, while elastomers contribute excellent elasticity, durability, and mechanical resilience. By addressing the inherent limitations of traditional bulk manufacturing methods, printing technologies offer precise spatial control, the ability to construct complex structures, and enhanced compatibility with biological systems. These capabilities are essential for the development of next-generation wearable and implantable bioelectronic devices. Despite the substantial progress achieved in this field, several challenges remain. These include the optimization of conductivity–mechanical property trade-offs, ensuring long-term biocompatibility and stability in vivo, and addressing the complex clinical and regulatory requirements. Continued interdisciplinary collaboration among materials science, bioengineering, and advanced manufacturing will be essential to fully realize the translational and clinical potential of printed soft bioelectronic systems.

## Data Availability

Not applicable.
